# Whey Protein Supplementation Combined with Exercise on Muscle Protein Synthesis and the AKT/mTOR Pathway in Healthy Adults: A Systematic Review and Meta-Analysis

**DOI:** 10.3390/nu17162579

**Published:** 2025-08-08

**Authors:** Xiaorong Ji, Xuanyin Ye, Shuyi Ji, Shuxin Zhang, Yuwen Wang, Zhibei Zhou, Dao Xiang, Beibei Luo

**Affiliations:** 1 Shanghai Key Laboratory of Human Performance, Shanghai University of Sport, Shanghai 200438, China; jixiaorong78@163.com (X.J.);; 2 School of Exercise and Health, Shanghai University of Sport, Shanghai 200438, China; 3 Center for Tumor Diagnosis & Therapy, Jinshan Hospital, Fudan University, Shanghai 201508, China; 4 Naval Medical Center, Naval Medical University, Shanghai 200433, China; 5 National Key Laboratory of Immunity and Inflammation, Naval Medical University, Shanghai 200433, China

**Keywords:** whey protein, exercise, muscle protein synthesis, AKT, mTOR

## Abstract

**Background:** The process of muscle protein synthesis (MPS) plays a pivotal role in the enhancement of muscle function. Following a bout of exercise, the rate of MPS experiences an elevation for a brief period, known as the “anabolic window.” Despite whey protein supplementation has been demonstrated to augment the post-exercise anabolic window, the optimal timing and dosage remain controversial. Therefore, the present systematic review and meta-analysis were conducted to evaluate the effects of whey protein supplementation on post-exercise MPS and its protein kinase B (AKT)/mammalian target of the rapamycin (mTOR) pathway in healthy adults. **Methods:** Following PRISMA guidelines, this review included 21 RCTs, with 15 studies subjected to meta-analysis and 6 studies to qualitative analysis. Eligible studies examined myofibrillar fractional synthetic rate (FSR) or the AKT/mTOR pathway-related protein phosphorylation levels in muscle biopsy samples. **Results:** The combination of whey protein supplementation and exercise has been shown to significantly enhance FSR (Hedge’s g = 1.24, 95% CI: 0.71–1.77; *p* < 0.001), with increases ranging from 1.3 to 1.6 folds when consumed immediately after exercise and up to 2.5 folds when given 45 min prior to multiple-set resistance exercise. A dose-dependent increase in FSR was observed in response to whey protein supplementation, ranging from 10 to 60 g. In comparison to the placebo group, whey protein supplementation enhanced the phosphorylation levels of AKT, mTOR, eukaryotic translation initiation factor 4E-binding protein-1 (4E-BP1), 70 kDa ribosomal protein S6 kinase (p70S6K), and ribosomal protein S6 (rpS6) at 1–2 h post-exercise. Phosphorylation levels of p70S6K and rpS6 decreased 4–5 h after exercise. **Conclusions:** The combination of whey protein supplementation and exercise improves MPS in a time- and dose-dependent manner. Consumption of 20–40g of whey protein before multiple sets of resistance exercise may enhance myofibrillar FSR and activate the AKT/mTOR pathway, thereby augmenting MPS and extending the anabolic window.

## 1. Introduction

Muscle protein synthesis (MPS) is fundamental to muscle function and serves as the structural basis for improvements in exercise performance. Following a bout of exercise, the rate of MPS experiences an elevation for a brief period, known as the “anabolic window” [1]. Research has demonstrated that exercise activates the protein kinase B/Mechanistic Target of Rapamycin (AKT/mTOR) signaling pathway [2]. This activation has been shown to stimulate mTOR1, thereby enhancing MPS efficiency and improving both short-term athletic ability and long-term muscle adaptation [3,4]. Moreover, an increase in MPS has been shown to facilitate muscle repair and mitigate post-exercise soreness and fatigue, thereby enhancing muscle strength, endurance, and anaerobic power recovery [5].

Whey protein is a prevalent sports nutrition supplement commonly employed in exercise training [6]. Meta-analyses have demonstrated that whey protein supplementation increases muscle and lean body mass in trained individuals, as well as significantly improving one-repetition maximum (1RM) [7,8]. Whey protein contains up to 26% branched-chain amino acids (BCAAs), and its leucine content is significantly higher than that of casein and soy protein. Due to its rapid digestion characteristics, plasma leucine concentrations reach peak levels shortly after ingestion, thereby conferring an advantage to whey protein in promoting MPS [9]. Leucine has been found to activate mTOR complex 1 (mTORC1) by modulating the activity of the mTOR signaling pathway and translation initiation factors. Subsequent to its activation, mTORC1 promotes the phosphorylation of the 70 kDa ribosomal protein S6 kinase (p70S6K) and 4E-binding protein 1 (4E-BP1), which ultimately leads to the phosphorylation of ribosomal protein S6 (rpS6) [10]. These phosphorylation events drive ribosomal assembly and mRNA translation initiation, directly stimulating MPS [11]. A study found that the AKT/mTOR pathway remains sensitive to leucine during overnight recovery after exercise [12].

The combination of exercise and whey protein supplementation has been demonstrated to enhance muscle capillary formation, vascular function, and antioxidant enzyme activation [13]. Moreover, the administration of whey protein following resistance exercise has been shown to significantly augment the activity and signaling of mTORC1 [14]. Exercise-induced signals and nutritional signals provided by protein supplementation have been shown to cross-talk and integrate, thereby regulating mTORC1 signaling and influencing protein synthesis rates in skeletal muscle [15]. The effect of combining exercise and protein supplementation on MPS is influenced by multiple factors, including pre-exercise nutritional status, total protein intake, and training experience [16].

Controversy remains regarding the optimal timing of and dosage of whey protein supplementation. Therefore, the aim of this review was to clarify the time-dependent pattern of whey protein supplementation combined with exercise on MPS and to explore optimal supplementation strategies.

## 2. Materials and Methods

This meta-analysis was conducted in accordance with the Preferred Reporting Items for Systematic Reviews and Meta-Analyses (PRISMA) guidelines. The analysis methodology and inclusion criteria were specified and documented in the registration protocol of the International Prospective Systems Evaluation Registry PROSPERO (CRD42024618933).

### 2.1. Eligibility Criteria

#### 2.1.1. Inclusion and Exclusion Criteria

The inclusion and exclusion criteria are outlined in Table 1. The following criteria were used to determine the inclusion of studies in the present analysis: (1) randomized controlled trials (RCTs); (2) written in English; (3) including healthy adults from non-sedentary populations; (4) whey protein supplements (hydrolyzed, concentrated or isolated) were used; (5) an acute, single-bout exercise intervention was performed; (6) MPS rate was assessed by muscle biopsy; and (7) the outcome measures included myofibrillar fractional synthetic rate (FSR) or phosphorylation levels of signaling pathway proteins related to MPS. The following types of literature will not be considered: (1) observational studies and review articles; (2) book chapters, conference abstracts, editorials, letters, and symposium materials; (3) studies involving non-human subjects; and (4) studies reporting on the effects of whey protein on other biological or physiological processes or diseases.

#### 2.1.2. Literature Search Strategy and Screening

A comprehensive literature search was conducted in the following electronic databases: PubMed, Web of Science, Embase and Scopus. The objective of the search was to investigate the effects of whey protein supplementation combined with exercise training on MPS rates and signaling pathways. Logical combinations were made using Boolean search operators and the following search terms: whey protein supplementation, resistance training, aerobic exercise, acute exercise, and muscle biopsy. The literature search was completed on 1/2025. The detailed literature search formula is provided in Supplementary Material Tables S1–S4.

It is imperative to note that all duplicate records were excluded from the analysis. The records were then subjected to independent screening by two reviewers. The screening process involved the evaluation of titles and abstracts against a set of predefined inclusion and exclusion criteria. Following the initial screening, the full text of the articles was subjected to a process of evaluation for its suitability for inclusion. The ultimate decision regarding the inclusion of a study was made by consensus between the two reviewers (X.J. and X.Y.). In instances of discordance regarding the inclusion of a particular article, a third reviewer (B.L.) was consulted to provide an adjudication.

### 2.2. Data Extraction

Following the literature screening process, relevant data from the included articles were extracted into Microsoft Excel 2019 (Microsoft, Redmond, WA, USA) by two members of the research team (X.J. and X.Y.) independently. The baseline population characteristics were extracted, including the sample sizes for each group, supplement composition and dosage, duration of supplementation, and exercise training regimen (i.e., frequency, intensity, and volume of training). Additionally, pre- and post-intervention measurements were taken. In instances where data were not available in the tables, text, or after multiple attempts to contact the authors, WebPlotDigitizer (version 4.6) was used to extract data from the plots.

### 2.3. Assessment of Risk of Bias

The quality and risk of bias in the included studies were assessed by two authors (X.J. and X.Y.) using the revised Cochrane collaboration tool for assessing risk of bias in randomized clinical trials (RoB, version 2.0). The present assessment was conducted independently and in a blind manner. The following five domains were assessed to evaluate the risk of bias of RCTs: the randomization process, deviation from intended interventions, missing outcome data, measurement of the outcome, and selection of reported results. The classification of each study was determined as low risk, high risk, or uncertain. The overall risk classification was determined by the study’s performance across all domains. The classification of a study as low risk was determined by the absence of bias across all domains, thereby ensuring the study’s reliability and credibility. In contrast, high or uncertain risk classifications were assigned to studies that exhibited potential biases or uncertainties in their methodologies, potentially compromising the trustworthiness of their findings. A study that was at high risk for only one domain, but not all domains, was classified as having raised some concerns. A study was classified as having a high risk of bias if it was at high risk in at least one domain or raised some concerns in multiple domains, thus substantially lowering confidence in the results. In instances of disagreement, the opinion of a third investigator (B.L.) was consulted to reach a consensus.

### 2.4. Meta-Analysis

The included studies were analyzed using R software version 4.3.1 (R Foundation for Statistical Computing, Vienna, Austria) by calculating the standardized mean difference (Hedge’s g) and the 95% confidence interval (CI). The extent of heterogeneity between studies was subjected to rigorous evaluation using the *I*^2^ statistic. Depending on the *I*^2^ result, either a fixed-effects model or a random-effects model was selected for the analysis. Specifically, if *I*^2^ exceeded 50%, indicating significant heterogeneity, a random-effects model was adopted to calculate the parameters. Conversely, in instances where substantial heterogeneity was absent (*I*^2^ ≤ 50%), the utilization of a fixed-effects model was assumed to be appropriate. A sensitivity analysis was performed for all outcomes based on the leave-one-out approach. In view of the limited number of selected studies, it was deemed inappropriate to utilize the funnel plot for assessing publication bias.

## 3. Results

### 3.1. Search Results

**Figure 1:**
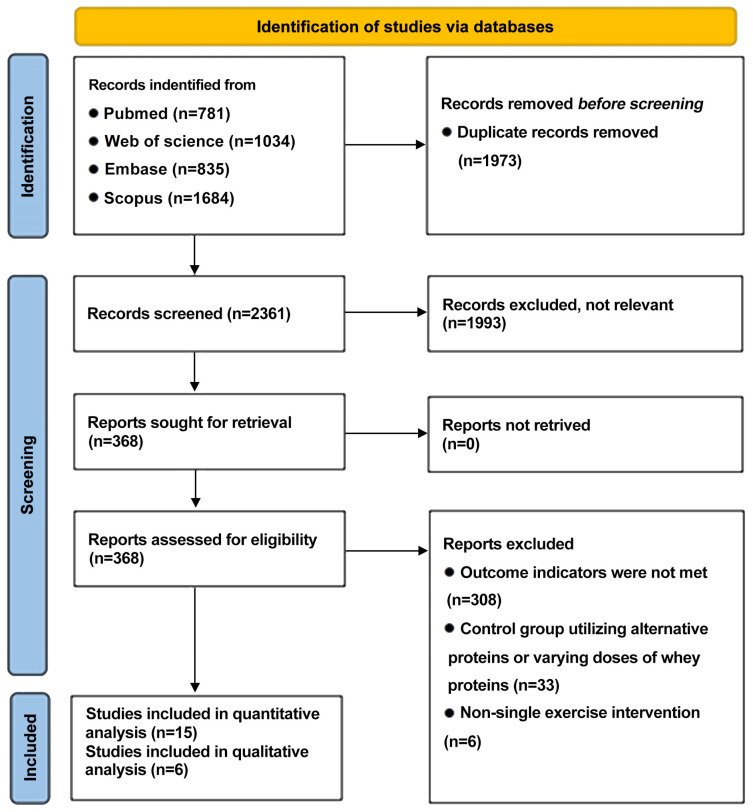
PRISMA flowchart outlining literature search screening and selection procedure.

A total of 4334 studies were identified through database searches, with 1973 remaining following the removal of duplicates. In the process of title and abstract screening, 1993 studies were excluded, resulting in 368 full-text articles that were assessed for eligibility. Following a thorough screening process that entailed the evaluation of the full texts, a total of 15 studies were selected for the final quantitative analysis, while 6 studies were selected for the qualitative analysis. The search procedure is summarized in Figure 1.

### 3.2. Study Characteristics

A total of 21 RCTs were included in this systematic review. The main characteristics of the included studies are shown in Table 2. Fourteen studies employed a parallel design, while the remaining seven utilized crossover design. The studies involved a total of 338 male participants and 33 female participants, with sample sizes ranging from 8 to 36 participants per study. The participants’ experience of exercise varied. A total of 17 studies incorporated participants who had experience with resistance training, with a frequency of at least 1 to 3 times per week. In contrast, four studies included participants with no exercise experience. All experimental groups received whey protein supplementation, with doses ranging from 10 g to 60 g per session. Some studies added supplements such as leucine, maltodextrin, and carbohydrates. The control groups were provided with a variety of beverages, including artificially sweetened water, noncaloric placebo drinks, carbohydrate-based drinks, soy protein, milk, and blend protein. The exercise protocols comprised a series of exercises, including leg press, leg extension, and knee extension exercises. These were typically performed at 70% to 80% of one-repetition maximum (1RM). Some studies incorporated additional exercises, such as cycling sprints, chest press, lat pulldown, and whole-body resistance training, which were performed to volitional exhaustion. The primary outcomes measured included myofibrillar FSR, which serves as a key quantitative indicator of MPS, and the activation of intracellular signaling pathways involved in MPS, including AKT, mTOR, p70S6K, 4E-BP1, and rpS6.

### 3.3. Qualitative Synthesis

Among the 21 included studies, 16 reported the effects of whey protein supplementation combined with exercise on myofibrillar FSR. Of these, 13 studies administered whey protein immediately after exercise [17,19,20,27,28,29,31,32,33,34,35,36,37]. Despite variations in exercise modalities and intensities, post-exercise FSR was consistently increased by 1.3–1.6 fold compared with the placebo. Three studies investigated the effects of whey protein supplementation before exercise on MPS [18,21,22]. In two of these studies, whey protein was consumed 30 min before exercise, which consisted of either ten 6 s cycling sprints or ten barbell back squat repetitions at 70% of 1RM. Compared with the control groups, myofibrillar FSR increased by 1.3 fold and 1.1 fold, respectively [21,22]. Notably, Burke et al. reported that supplementation 45 min prior to eight sets of ten repetitions of leg extension at 80% 1RM increased FSR by 2.5 fold compared with the placebo [18].

Three studies investigated the effects of different doses of whey protein on myofibrillar FSR. Witard et al. reported that supplementation with 10 g, 20 g, and 40 g of whey protein increased the FSR by 1.2, 1.4, and 1.5 fold, respectively, compared with placebo [31]. Macnaughton et al. found that, under an identical exercise protocol, immediate post-exercise supplementation with 40 g of whey protein resulted in a 1.2-fold higher FSR compared with 20 g [34]. Another study conducted with female participants showed that supplementation with 15 g, 30 g, and 60 g of whey protein immediately after exercise increased the FSR by 1.5, 2.0, and 1.5 fold compared to the baseline, respectively [35].

Furthermore, four studies compared the effects of whey protein with other protein sources. Churchward-Venne et al. demonstrated that whey and soy protein led to similar myofibrillar FSR responses [32]. One study found that post-exercise ingestion of whey protein or milk increased the myofibrillar FSR by 2.3 fold and 0.9 fold compared to the baseline, respectively [33]. Another study showed that immediate post-exercise ingestion of whey, soy, or casein protein increased the myofibrillar FSR by 1.7, 1.5, and 1.4 fold compared to the baseline, respectively [37]. Reidy et al. reported that a blended protein supplement consisting of whey, soy, and casein increased the FSR to a greater extent than whey protein alone [36]. These findings suggest that whey protein is more effective than soy, casein, and milk in promoting myofibrillar protein synthesis following exercise and that multi-protein blends may further enhance this effect, whereas whole milk is less effective and not recommended.

### 3.4. Meta-Analysis Results

#### 3.4.1. Myofibrillar FSR

**Figure 2:**
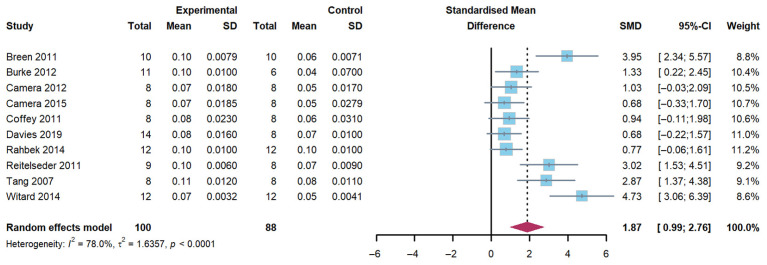
Effects on myofibrillar fractional synthetic rates [17,18,19,20,21,22,27,28,29,31].

A total of 10 studies were included in the evaluation of the effect of whey protein supplementation on myofibrillar FSR, with measurements taken 3 to 5 h post-exercise. The findings demonstrated that the combination of exercise and whey protein supplementation resulted in a significant increase in myofibrillar FSR compared to exercise alone (Hedge’s g of 1.87; 95% CI: 0.99 to 2.76; *p* < 0.001). The heterogeneity (*I*^2^) statistic was 78.0% (Figure 2).

#### 3.4.2. Protein Phosphorylation Level of AKT/mTOR Pathway

An investigation was conducted into the pivotal signaling pathways involved in MPS, with a particular focus on AKT, mTOR, 4E-BP1, rpS6, and p70S6K, at two distinct post-exercise time points. The duration of the first period was between 1 and 2 h, and the duration of the second period was between 4 and 5 h, with the aim of determining whether exercise combined with whey protein supplementation further enhances the activity of signaling pathways related to MPS compared to exercise alone.

##### AKT

**Figure 3:**
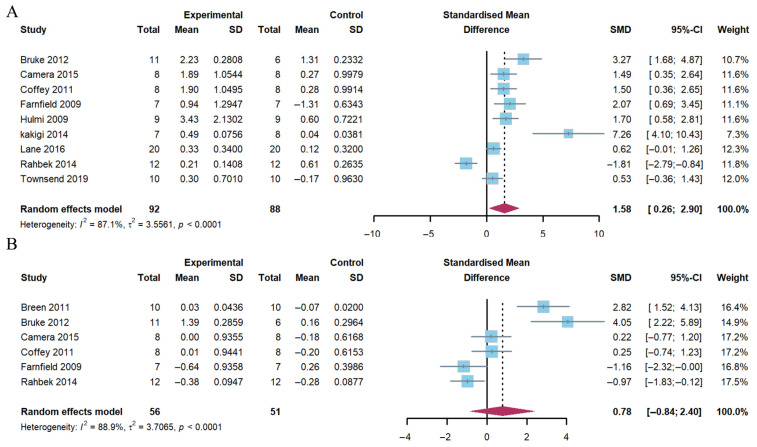
Effects on AKT phosphorylation at 1–2 h (**A**) and 4–5 h (**B**) post-exercise [17,18,20,21,23,24,25,26,27,29].

The pooled analysis revealed a significant increase in AKT phosphorylation 1–2 h following exercise (Hedge’s g of 1.58; 95% CI: 0.26 to 2.90; *p* = 0.005) in the group with a combination of exercise and whey protein supplementation. The heterogeneity (*I*^2^) was 87.1%. However, no differences were observed at 4–5 h post-exercise (Hedge’s g of 0.78; 95% CI: −0.84 to 2.40; *p* = 0.284). The heterogeneity (*I*^2^) was 88.9% (Figure 3).

##### mTOR

**Figure 4:**
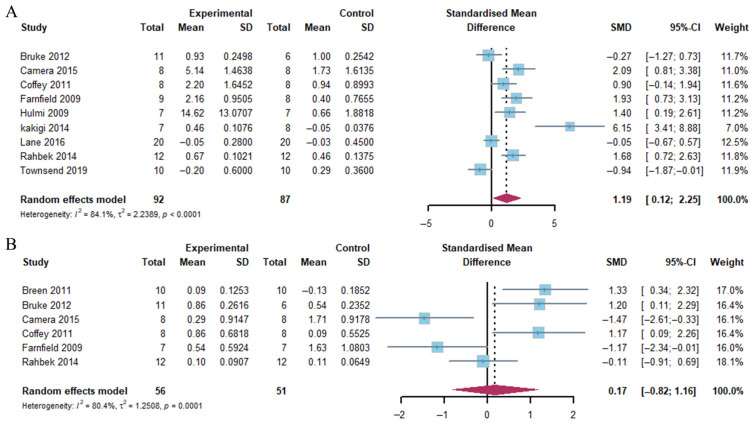
Effects on mTOR phosphorylation at 1–2 h (**A**) and 4–5 h (**B**) post-exercise [17,18,20,21,23,24,25,26,27,30].

The present analysis demonstrated that the combination of exercise and whey protein supplementation resulted in a notable increase in mTOR phosphorylation 1–2 h post-exercise (Hedge’s g = 1.19; 95% CI: 0.12 to 2.25; *p* = 0.014). The observed heterogeneity (*I*^2^) was 84.1%. However, no alterations were identified at 4–5 h post-exercise (Hedge’s g = −0.17; 95% CI: −0.82 to 1.16; *p* = 0.720), with heterogeneity (*I*^2^ = 80.4%) (Figure 4).

##### E-BP1

**Figure 5:**
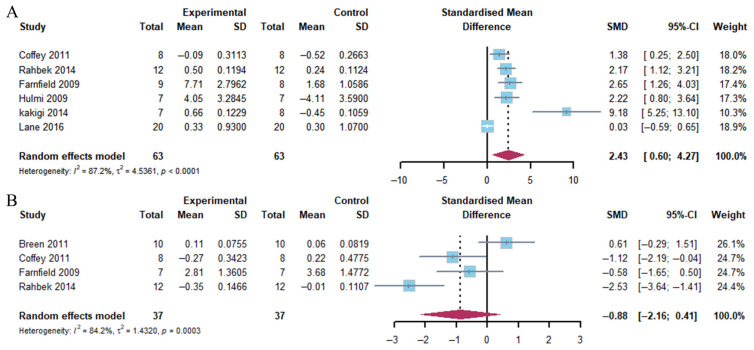
Effects on 4E-BP1 phosphorylation at 1–2 h (**A**) and 4–5 h (**B**) post-exercise [17,21,23,24,25,26,27].

The results of the pooled analysis showed a marked increase in 4E-BP1 phosphorylation 1–2 h after exercise, particularly in the group with a combination of exercise and whey protein supplementation (Hedge’s g = 2.43; 95% CI: 0.60 to 4.27; *p* = 0.002). The heterogeneity of the data was found to be substantial (*I*^2^ = 87.2%). In contrast, 4E-BP1 phosphorylation exhibited no significant change in response to the combination of exercise and whey protein supplementation at 4–5 h post-exercise (Hedge’s g = −0.88; 95% CI: −2.16 to 0.41; *p* = 0.188) (Figure 5). This outcome demonstrated moderate heterogeneity (*I*^2^ = 84.2%).

##### p70S6K

**Figure 6:**
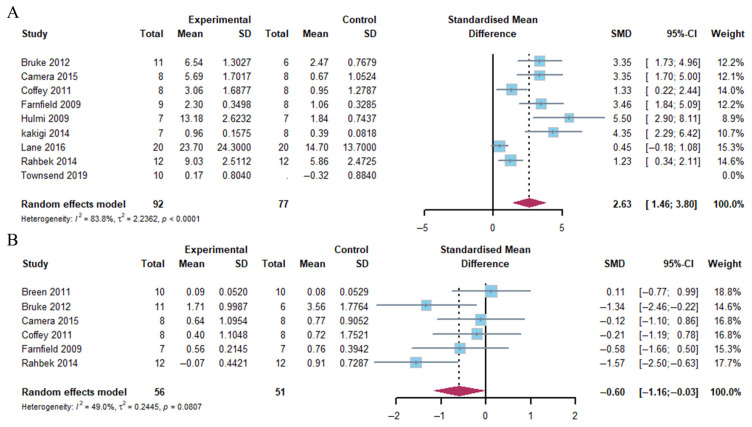
Effects on p70S6K phosphorylation at 1–2 h (**A**) and 4–5 h (**B**) post-exercise [17,18,20,21,23,24,25,26,27,30].

The present analysis revealed that the combination of exercise and whey protein supplementation led to a pronounced augmentation in p70S6K phosphorylation 1–2 h after exercise, as compared to exercise alone (Hedge’s g = 2.63, 95% CI: 1.46 to 3.80; *p* < 0.001). The heterogeneity of the data was found to be high (*I*^2^ = 83.8%). However, at 4–5 h post-exercise, p70S6K phosphorylation exhibited a marked decrease with the combination of exercise and whey protein supplementation (Hedge’s g = −0.60; 95% CI: −1.16 to −0.03; *p* = 0.049), accompanied by low heterogeneity (*I*^2^ = 49.0%) (Figure 6).

##### rpS6

**Figure 7:**
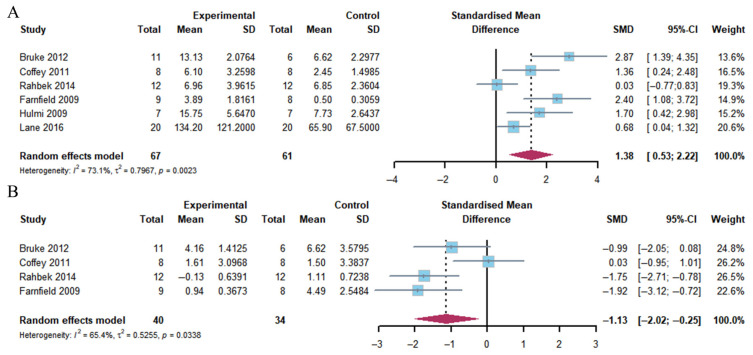
Effects on rpS6 phosphorylation at 1–2 h (**A**) and 4–5 h (**B**) post-exercise [18,21,23,24,26,27].

Our meta-analysis demonstrated that a considerable growth in rpS6 phosphorylation was evident within the first two hours following exercise in response to the combination of exercise and whey protein supplementation (Hedge’s g = 1.38; 95% CI: 0.53 to 2.22; *p* = 0.001). The heterogeneity of the data was moderate (*I*^2^ = 73.1%). In contrast, at 4–5 h post-exercise, rpS6 phosphorylation exhibited a marked decrease in response to the combination of exercise and whey protein supplementation (Hedge’s g = −1.13; 95% CI: −2.02 to −0.25; *p* = 0.013), accompanied by moderate heterogeneity (*I*^2^ = 65.4%) (Figure 7).

#### 3.4.3. Sensitivity Analysis

To guarantee the validity of the findings, a sensitivity analysis was conducted. This involved the sequential exclusion of one study at a time. This analysis revealed that the direction and magnitude of the pooled estimates for muscle protein fractional synthesis and the phosphorylation of proteins involved in protein synthesis pathways (AKT, mTOR, 4E-BP1, p70S6K and rpS6) remained stable across all exclusions (Figures S1–S6). The findings of the present study indicate that the overall results were not substantially affected by the exclusion of any individual study.

### 3.5. Risk of Bias

**Figure 8:**
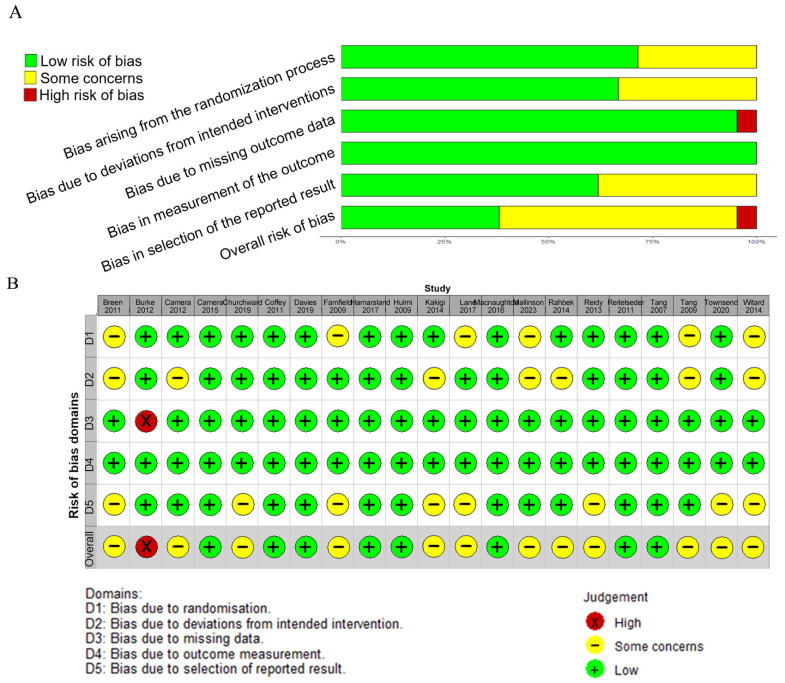
Risk of bias (**A**) and the summary of risk of bias (**B**) for the included studies [17,18,19,20,21,22,23,24,25,26,27,28,29,30,31,32,33,34,35,36,37].

The quality of the studies included varied from low to high risk of bias. Among the 21 studies that were included in the analysis, 1 was assessed as having a “high risk” of bias overall, 12 were rated as having “some concerns,” and 8 were considered to have a “low risk” of bias. In terms of randomization, 14 studies were classified as low risk, while 4 raised some concerns. Regarding deviations from intended interventions, 5 studies raised some concerns and 13 were rated as low risk. For missing outcome data, 17 studies were found to be at low risk, while 1 was at high risk. For the risk of bias in the outcome measurement, all studies were classified as low risk. Regarding the selection of the reported results, 5 studies were found to raise some concerns and 13 were considered to be at low risk (Figure 8).

## 4. Discussion

**Figure 9:**
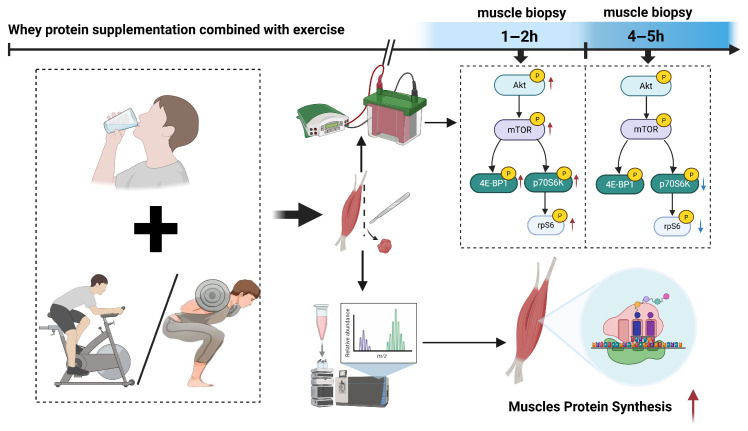
The findings of this systematic review and meta-analysis indicate that whey protein supplementation combined with exercise significantly enhances MPS at 3–5 h post-exercise. Concurrently, this intervention potently activates key components of the AKT/mTOR signaling pathway (including AKT, mTOR, 4E-BP1, p70S6K, and rpS6), with phosphorylation levels of p70S6K and rpS6 peaking at 1–2 h post-exercise. Subsequently, these phosphorylation markers demonstrate a decline, typically returning towards baseline by 4–5 h post-exercise. Red arrow: upregulated. Blue arrow: downregulated.

The present systematic review and meta-analysis demonstrate that the ingestion of whey protein in conjunction with physical exercise results in a substantial increase in MPS by 3 to 5 h post-exercise (Figure 9). The phosphorylation levels of the AKT/mTOR pathway and its downstream targets 4E-BP1, rpS6, and p70S6K increased at 1–2 h post-exercise. The phosphorylation levels of AKT and mTOR returned to baseline, while the rpS6 and p70S6K levels decreased by 4–5 h post-exercise.

### 4.1. The Effects of Whey Protein Supplementation on MPS

Whey protein has been extensively utilized among athletes to enhance muscle function and exercise performance [38]. As asserted by the International Society of Sports Nutrition, the recommended daily protein intake for athletes is within the range of 1.4 to 2.0 g/kg/d [39]. Whey protein is rapidly broken down in the digestive tract, causing plasma amino acid levels to reach a high level 100 min after ingestion., particularly leucine [40]. Leucine constitutes the primary element of whey protein and is regarded as a pivotal branched-chain amino acid within this protein. It fulfills a regulatory function in muscle metabolic homeostasis. The rapid absorption characteristics of whey protein enable it to efficiently promote muscle synthesis during the “anabolic window” period after exercise, when muscles are most sensitive to protein [41]. Leucine supplements have been demonstrated to stimulate protein synthesis by activating the mTORC1 signaling pathway [42]. The synergistic effect of leucine and insulin has been shown to enhance the net balance of protein synthesis and catabolism in skeletal muscle [43]. Leucine-mediated activation of the mTOR pathway is influenced by basal p70S6K levels, the intensity of insulin signaling, and the associated negative feedback regulation [44,45].

### 4.2. The Role of the AKT/mTOR Pathway in the Regulation of MPS

The AKT/mTOR pathway represents a pivotal molecular network that regulates protein synthesis in skeletal muscle. The activity of the AKT/mTOR pathway is subject to regulation by a variety of factors, including nutrients, growth factors, subcellular localization, energy status, and stress responses [46,47,48,49]. The AKT/mTOR pathway is involved in the prevention of muscle atrophy induced by disuse [50]. The application of mechanical tension through exercise stimulates the AKT through the insulin-like growth factor 1 (IGF-1)/phosphatidylinositol 3-kinase (PI3K) pathway, thereby facilitating the inactivation of tuberous sclerosis complex 2 (TSC2) phosphorylation and increasing mTORC1 activity [51]. Leucine, a constituent of whey protein, has been demonstrated to rapidly activate mTORC1 by binding to sestrin2, thereby releasing its inhibition of GTPase-activating protein towards Rags 2 (GATOR2) [52]. A study has demonstrated that whey protein supplementation exerts a synergistic activating effect on the AKT/mTOR pathway in response to exercise-induced mechanical tension signaling [53]. Furthermore, mTORC1 activation was followed by the inhibition of PI3K signaling by p70S6K. In addition, the sustained activation of p70S6K has been shown to induce feedback inhibition of TSC2 [54].

The current review indicated that the phosphorylation levels of the AKT/mTOR downstream signaling proteins 4E-BP1, rpS6, and p70S6K peaked at 1–2 h post-exercise, whereas the significant increase in MPS was delayed until 3–5 h post-exercise. Furthermore, the rapid return of pathway activity to baseline by 4–5 h despite sustained MPS elevation suggests that late phase signaling may not be essential for maintaining protein synthesis. 4E-BP1 has been identified as the primary negative regulator of eIF4E. Phosphorylation of 4E-BP1 has been shown to trigger the release of eIF4E [55]. A study has shown that the phosphorylation status of eIF4E functions as a key regulator of MPS. Phosphorylated eIF4E has been shown to be capable of overcoming the inhibition of 4E-BP1, thus facilitating the initiation of the translation process and promoting MPS [56]. Meanwhile, p70S6K, a key kinase downstream of mTORC1, has been shown to promote ribosomal biosynthesis by phosphorylating ribosomal protein S6 (rpS6) [57]. Phosphorylation of rpS6 is associated with ribosome biogenesis and selective translation [58,59]. These processes support muscle protein synthesis [11]. However, the mechanisms by which these effects occur require still need further validation. A recent study using transcriptome gene expression sequencing revealed that the mRNA of PIK3R3, a key subunit of PI3K, increased one hour after high-intensity resistance exercise and remained elevated above baseline levels four hours post-exercise [60]. These results are in alignment with the temporal characteristics of signaling protein activation observed.

### 4.3. Exercise Interventions on Whey Protein Supplementation Efficacy

The most significant effect was observed in the group that underwent resistance training in combination with whey protein supplementation. A study demonstrated that HIIT activates AMPK to promote mitochondrial biosynthesis, in contrast to its weaker acute effect on MPS [61]. The dose of whey protein, the timing of ingestion, and differences in the type, intensity, and volume of exercise have the potential to influence the level of activation of signaling pathways [62]. One study revealed that individuals lacking prior training experience exhibited a more pronounced increase in MPS compared to those with experience in resistance training [28]. Another study has shown that the effects of protein supplementation on enhancing muscle strength and size in healthy adults during prolonged resistance training diminish with increasing training levels [8]. In the present review, only one study utilized a HIIT exercise intervention. There was no significant difference in the effects on MPS compared to RE studies [21]. A recent study administered the ingestion of 10 g of whey protein at 20 min intervals over a continuous period and found that the levels of rpS6 phosphorylation were significantly elevated by 85% compared to the placebo, as early as 120 min after exercise, under conditions of whey protein intake. This increase in phosphorylation levels persisted until 300 min had elapsed [63].

The ingestion of protein during this period has been shown to enhance muscle repair and growth [41]. Among the 15 RCTs included in this meta-analysis, 11 included supplementation after exercise [17,19,20,23,25,26,27,28,29,30,31], 3 included supplementation before exercise [18,21,22], and 1 included supplementation both before and after exercise [24]. The results showed no difference in the effect on MPS. Consequently, it is proposed that exercise-bound whey protein promotes a wider window of protein synthesis than was previously hypothesized (30 min to 2 h) and that its window of action may extend up to 12 h post-exercise [64]. In particular, the synergistic supplementation of proteins with carbohydrates has the potential to further optimize MPS. One study added fructose to whey protein supplementation, and intermediates such as fructose-6-phosphate, which is produced by the metabolism of fructose in glycolysis, may be utilized in anabolic processes via a similar mechanism, thus supporting MPS [65].

### 4.4. The Impact of Whey Protein Supplementation on Muscle Function and Athletic Performance

The ingestion of dietary protein supplements has been demonstrated to have a substantial impact on the alterations in muscular strength and size that occur during extended periods of resistance training in healthy adults [8]. For trained individuals, whey protein supplementation has been shown to significantly increase muscle and lean body mass, as well as 1RM [7,8]. Research has found that milk proteins, encompassing milk, whey protein, yogurt, casein, and bovine colostrum, have been demonstrated to be efficacious in promoting gains in muscle mass and strength. Some studies have also found that whey protein supplementation led to the most consistent gains in muscular strength compared to other protein sources [66]. Our review found that whey protein supplementation was more effective than milk at improving MPS and that a mixture of multiple proteins was even more effective [33,36].

Our review demonstrated that the combination of whey protein supplementation and exercise training significantly enhances post-exercise MPS. A six-week 25 g/day whey protein supplementation and 4000 IU vitamin D3 supplements, in conjunction with a resistance training regimen, elicited significant improvements in muscle mass and strength in healthy young males [67]. Acute 30 g/day whey protein and omega-3 supplementation, particularly before exercise, improves strength and power and reduces delayed-onset muscle soreness in female futsal players [68]. A 4-week study found that 40 g/day of whey protein in combination with three times/week RE significantly increases lean body mass, as well as improving iso-kinetic strength and muscular endurance of the knee and shoulder joints [69]. The ingestion of protein in quantities exceeding 1.6 g per day per kilogram of body weight does not appear to promote additional increases in fat-free mass resulting from exercise [8]. Therefore, when combined with structured resistance or eccentric training, it improves MPS and leads to other outcomes such as improved strength, power, and recovery.

### 4.5. Study Limitations and Future Directions

Despite all the studies being RCTs, the heterogeneity in the experimental designs, such as crossover versus parallel formats, and in the sample sizes, ranging from 8 to 22 participants, may explain the intervention effect variations. Despite the utilization of random-effects models to address heterogeneity, the elevated *I*^2^ values (>50%) signify considerable disparities among the trials. Therefore, the interpretation of pooled effect sizes should be approached with caution. Future studies should be conducted with expanded sample sizes, optimized experimental designs, and adapted intervention strategies to enhance the reliability of the findings.

In addition, the metabolic status of the participants, including insulin sensitivity and basal amino acid levels, as well as gender differences influenced by sex hormones, may further affect the effectiveness of the intervention [70,71,72]. The present review included 33 female participants, with male participants still accounting for the majority. It has been demonstrated that androgen deficiency results in reduced activation of the AKT/mTOR pathway, consequently lowering MPS [73]. Due to discrepancies in hormones and other variables, male and female subjects will undergo divergent physiological changes under analogous experimental conditions or intervention factors. Consequently, the conclusions of the present study may not be universally applicable to the female population. It is further anticipated that subsequent research endeavors will dedicate more attention to female populations. This will provide new insights into the mechanisms by which hormones regulate physiological functions, including muscle synthesis. Furthermore, it is expected to yield novel concepts for addressing gender-related challenges in practice.

A decline in muscle synthesis capacity has become evident among individuals aged 50 and above [74]. Individuals aged 45 and above are also susceptible to an elevated risk of developing sarcopenia [75]. Subsequent research endeavors should prioritize the study of adolescents and middle-aged individuals. This focus would yield novel insights into the domain of MPS-related fields and facilitate the development of practical solutions for addressing issues associated with population aging.

With regard to the experimental design of the included studies, there were significant variations in the dosage of whey protein supplements and the timing of intake across the studies, with only two articles comparing different dosages. Therefore, there is a need to expand the study population and further elucidate the potential mechanisms of whey protein supplementation and exercise interventions. This can be achieved by comparing different types of exercise intervention strategies. To optimize the utilization of whey protein and exercise in enhancing MPS, it is recommended that future studies focus on the development of customized whey protein supplementation regimens and the design of precise exercise protocols.

## 5. Conclusions

Whey protein supplementation combined with exercise improves MPS at 3–5 h post-exercise. This effect was achieved through the time-dependent activation of the AKT/mTOR signaling pathway, involving 4E-BP1, rpS6, and p70S6K, which increased within 1–2 h before reverting to baseline by 4–5 h. The consumption of 20–40 g of whey protein before multiple sets of resistance exercise may enhance myofibrillar FSR, thereby augmenting MPS and extending the anabolic window.

## Figures and Tables

**Table 1:** Selection criteria.

Category	Inclusion Criteria	Exclusion Criteria
Population	Healthy adults (18–50 years) from non-sedentary populations.	Sedentary individuals, the elderly, and people with chronic diseases.
Intervention	Whey protein supplements (hydrolyzed, concentrated, or isolated) are used in combination with acute single-bout exercise intervention.	Long-term exercise intervention combined with non-whey protein supplementation.
Comparator	The control groups receive various alternatives, including artificially sweetened water, noncaloric placebo drinks, carbohydrate-based drinks, soy protein, milk, and blend protein.	—
Outcome	Outcome measures include myofibrillar FSR or phosphorylation levels of signaling pathway proteins related to MPS.	Fails to assess MPS or lacks quantifiable phosphorylation data of signaling pathway proteins related to MPS.
Study design	The study protocol is RCT with a parallel or crossover design.	Observational studies and review articles.

**Table 2:** Summary of the intervention protocols included in the meta-analysis and systematic review.

Study	Study Type	Subject (Sample Size, Gender, Age, Training Experience)	Exercise Protocol	Supplementary Protocol	Supplement Timing	Outcomes	
						FSR (Fold Changes)	Post-Exercise Phosphorylation Levels of AKT/mTOR Pathway (Ex vs. Con)
Breen et al. 2011 [17]	RCT (crossover)	10, male, 29.0 ± 6.0 years, >2 times/week for 7.5 ± 3.0 years	90 min cycling exercise at 75% Wmax	Ex: Carbohydrate (25.4 g) × 2 + whey protein (10.2 g) × 2; Con: Carbohydrate (25.2 g) × 2	Immediately and 30 min post-exercise	1.6 (Ex vs. Con)	4–5 h: AKT↑, mTOR↑, 4E-BP1↑, p70S6k↑
Burke et al. 2012 [18]	RCT (crossover)	12, male, 27.0 ± 1.3 years, 2 times/week for >2 years	10 sets, 8–10 repetitions of leg extension at 80% 1RM	Ex: Whey protein (25 g) + leucine (5 g); Con: Artificially sweetened water	45 min pre-exercise	2.5 (Ex vs. Con)	1–2 h: AKT↑, mTOR↓, p70S6K↑, rpS6↑ 4–5 h: AKT↑, mTOR↑, p70S6k↓, rpS6↓
Camera et al. 2012 [19]	RCT (parallel)	16, male, 22.9 ± 2.6 years, 3 times/week for >1 year	8 sets, 5 repetitions of leg press at 80% 1RM	Ex: Whey protein (20 g) ×2 + maltodextrin (40 g) × 2; Con: Artificially sweetened water × 2	Immediately and 2 h post-exercise	1.4 (Ex vs. Con)	—
Camera et al. 2015 [20]	RCT (crossover)	8, male, 19.1 ± 1.4 years, 3 times/week for >1 year	8 sets, 5 repetitions of leg extension at 80% 1RM	Ex: Whey protein (25 g); Con: Artificially sweetened water	Immediately post-exercise	1.4 (Ex vs. Con)	1–2 h: AKT↑, mTOR↑, p70S6K↑ 4–5 h: AKT↑, mTOR↓, p70S6k↓
Coffey et al. 2011 [21]	RCT (crossover)	8, male, 21.4 ± 2.6 years, 4–6 times/week	10 sets, 6 s cycling sprints at 0.75 N·m/kg	Ex: Whey protein (24 g) + additional leucine (4.8 g) + maltodextrin (50 g); Con: Artificially sweetened water	30 min pre-exercise	1.3 (Ex vs. Con)	1–2 h: AKT↑, mTOR↑, 4E-BP1↑, p70S6K↑, rpS6↑ 4–5 h: AKT↑, mTOR↑, 4E-BP1↓, p70S6k↓, rpS6↑
Davies et al. 2019 [22]	RCT (parallel)	22, male, 23.0 ± 4.3 years, >3 h/week for >6 months	10 repetitions of barbell back squats at 70% 1RM until failure to complete a full set	Ex: Whey protein (0.33 g/kg); Con: Nonessential amino acids	30 min pre-exercise	1.1 (Ex vs. Con)	—
Farnfield et al. 2009 [23]	RCT (parallel)	14, male, 22.5 ± 1.0 years, no training experience	3 sets, 12 repetitions of maximal single-leg knee extension	Ex: Whey protein isolated (26.6 g); Con: Placebo drink	Immediately post-exercise	—	1–2 h: AKT↑, mTOR↑, 4E-BP1↑, p70S6K↑, rpS6↑ 4–5 h: AKT↓, mTOR↓, 4E-BP1↓, p70S6k↓, rpS6↓
Hulmi et al. 2009 [24]	RCT (parallel)	18, male, 24.9 ± 3.8 years, no training experience	5 sets, 10 repetitions of leg press at 75% 1RM	Ex: Whey protein (15 g) × 2; Con: Nonenergetic placebo drink × 2	Immediately pre- and post-exercise	—	1–2 h: AKT↑, mTOR↑, 4E-BP1↑, p70S6K↑, rpS6↑
Kakigi et al. 2014 [25]	RCT (parallel)	15, male, 22.3 ± 0.3 years, no training experience	4 sets, 6 repetitions of maximal knee extension	Ex: Whey protein (10/20 g); Con: Water	Immediately post-exercise	—	1–2 h: AKT↑, mTOR↑, 4E-BP1↑, p70S6K↑
Lane et al. 2017 [26]	RCT (crossover)	20, male, 27.8 ± 2.8 years, 2–10 h/week	5 sets, 10 repetitions of leg press and leg extension at 10RM	Ex: Whey protein (10 g) + leucine (10 g); Con: Carbohydrates (4 g)	Immediately post-exercise	—	1–2 h: AKT↑, mTOR↓, 4E-BP1↑, p70S6K↑, rpS6↑
Rahbek et al. 2014 [27]	RCT (parallel)	22, male, 23.9 ± 0.8 years, ≥1 time/2 week for >6 months	6 sets, 10 repetitions of maximal knee extension	Ex: Whey protein hydrolysate (0.3 g/kg) + carbohydrate (0.3 g/kg); Con: Carbohydrate (0.60 g/kg)	Immediately post-exercise	1 (Ex vs. Con)	1–2 h: AKT↓, mTOR↑, 4E-BP1↑, p70S6K↑, rpS6↑ 4–5 h: AKT↓, mTOR, 4E-BP1↓, p70S6k↓, rpS6↓
Reitelseder et al. 2011 [28]	RCT (parallel)	17, male, 27.1 ± 2.2 years, no training experience	10 sets, 8 repetitions of leg extension at 80% 1RM	Ex: Whey protein (0.3 g/kg); Con: Noncaloric control drink	Immediately post-exercise	1.4 (Ex vs. Con)	—
Tang et al. 2007 [29]	RCT (crossover)	8, male, 21.0 ± 1.0 years, ≥3 times/week	4 sets, 8–10 repetitions of leg extension and leg press at 80% 1RM	Ex: Whey protein isolated (10 g) + fructose (21 g); Con: Fructose (21 g) + maltodextrin (10 g)	Immediately post-exercise	1.3 (Ex vs. Con)	—
Townsend et al. 2020 [30]	RCT (crossover)	10, male, 24.4 ± 4.1 years for >1 years	4 sets, 8–10 repetitions of leg press and leg extension at 75% 1RM	Ex: Whey protein (26 g); Con: Noncaloric control drink	Immediately post-exercise	—	1–2 h: AKT↑, mTOR↓, p70S6K↑
Witard et al. 2014 [31]	RCT (parallel)	24, male, 22.0 ± 3.0 years, ≥6 months	8 sets, 10 repetitions of leg extension at 80% 1RM	Ex: Whey protein (10 g/20 g/40 g) Con: Noncaloric control drink	Immediately post-exercise	1.2 (10 g vs. Con), 1.4 (20 g vs. Con), 1.5 (40 g vs. Con)	—
Churchward-Venne et al. 2019 [32]	RCT (parallel)	36, male, 23.0 ± 0.4 years, 3 times/week	4 sets, 8 repetitions of leg press at 80% 1RM + 30 min cycling exercise at 60% Wmax	Whey protein (20 g), Soy protein(20 g), Soy protein + leucine(20 g)	Immediately post-exercise	1 (Whey protein vs. Soy protein), 1 (Whey protein vs. Soy protein + leucine)	—
Hamarsland et al. 2017 [33]	RCT (parallel)	13, male, 9, female, 25.0 ± 3.8 years, >1 time/week for >6 months	4 sets, 8 repetitions of leg press and knee extension at 8RM	Whey protein (20 g) × 2, Milk (20 g) × 2, WPC-80 (20 g) × 2	Immediately and 2 h post-exercise	Compared with baseline: 0.9 (Milk), 2.3 (Whey protein), 2.4 (WPC-80)	—
Macnaughton et al. 2016 [34]	RCT (parallel)	30, male, 22.3 ± 3.0 years, ≥2 times/week for >6 months	3 sets, 10 repetitions of chest press, lat pulldown, leg curl, leg press and leg extension at 75% 1RM	Whey protein (20 g/40 g)	Immediately post-exercise	1.2 (40g vs. 20g)	—
Mallinson et al. 2023 [35]	RCT (parallel)	24, female, 26.6 ± 0.8 years, ≥2 times/week for >6 months	3 sets, 8 repetitions of lat pulldown, single-leg press on both legs and chest press at 75% 1RM	Whey protein (15 g/30 g/60 g)	Immediately post-exercise	Compared with baseline: 1.5 (15 g), 2.0 (30 g), 1.5 (60 g)	—
Reidy et al. 2013 [36]	RCT (parallel)	17, male, 24.1 ± 1.5 years, 2 times/week	8 sets, 10 repetitions of leg extension at 55–70% 1RM	Whey protein (17.7 g), Blend protein (19.3 g)	1 h post-exercise	Compared with baseline: 1.3 (Whey protein), 1.5 (Blend)	—
Tang et al. 2009 [37]	RCT (parallel)	18, male, 22.8 ± 3.9 years, 2–3 times/week	4 sets, leg press and leg extension at 10–12RM	Whey protein (21.4 g), Soy protein (22.2 g), Casein (21.9 g)	Immediately post-exercise	Compared with baseline: 1.7 (Whey protein), 1.5 (Soy protein), 1.4(Casein)	—

RCT: randomized controlled trial; RM: repetition maximum; FSR: fractional synthetic rate; AKT: protein kinase B; mTOR: mammalian target of rapamycin; Ex: experimental group; Con: control group; WPC-80: whey protein concentrate; 4E-BP1: 4E-binding protein-1; p70S6K: 70 kDa ribosomal protein S6 kinase; rpS6: ribosomal protein S6; ↑: increased; ↓: decreased.

## Data Availability

The data is available on request from the corresponding author.

## Supplementary Materials

The following supporting information can be downloaded at https://www.mdpi.com/article/10.3390/nu17162579/s1. Table S1: Search strategy in PubMed. Table S2: Search strategy in Web of Science. Table S3: Search strategy in Embase. Table S4: Search strategy in Scopus. Figure S1: Sensitivity analysis of myofibrillar fractional synthetic rates. Figure S2: Sensitivity analysis of AKT phosphorylation at 1–2 h (A) and 4–5 h (B) post-exercise. Figure S3: Sensitivity analysis of mTOR phosphorylation at 1–2 h (A) and 4–5 h (B) post-exercise. Figure S4: Sensitivity analysis of 4E-BP1 phosphorylation at 1–2 h (A) and 4–5 h (B) post-exercise. Figure S5: Sensitivity analysis of p70S6K phosphorylation at 1–2 h (A) and 4–5 h (B) post-exercise. Figure S6: Sensitivity analysis of rpS6 phosphorylation at 1–2 h (A) and 4–5 h (B) post-exercise.
