# Supplementary material for: Whey Protein Supplementation Combined with Exercise on Muscle Protein Synthesis and the AKT/mTOR Pathway in Healthy Adults: A Systematic Review and Meta-Analysis
Source: Nutrients. 2025 Aug 8;17(16):2579. doi: 10.3390/nu17162579 (PMC12389377; doi:10.3390/nu17162579)
Supplement: Supplementary file 1 [file nutrients-17-02579-s001.zip › nutrients-3758353-supplementary.pdf]

Table S1-S4 Search strategy

Table S1. Search strategy in PubMed.

Search	Query
#1	((((((((((((((whey proteins[MeSH Terms]) OR (whey[Title/Abstract])) OR (whey protein[Title/Abstract])) OR (WP[Title/Abstract])) OR (whey supplementation[Title/Abstract])) OR (whey protein supplementation[Title/Abstract])) OR (whey intake[Title/Abstract])) OR (WPI[Title/Abstract])) OR (whey protein isolate[Title/Abstract])) OR (WPC[Title/Abstract])) OR (whey protein concentrate[Title/Abstract])) OR (milk proteins[Title/Abstract])) OR (milk protein[Title/Abstract])) OR (dairy proteins[Title/Abstract])) OR (dairy protein[Title/Abstract])
#2	((((((((((((((((((((((Exercise[MeSH Terms])) OR (Exercises[Title/Abstract])) OR (Exercise, Physical[Title/Abstract])) OR (Exercises, Physical[Title/Abstract])) OR (Physical Activity[Title/Abstract])) OR (Activities, Physical[Title/Abstract])) OR (Activity, Physical[Title/Abstract])) OR (Physical Activities[Title/Abstract])) OR (Aerobic Exercise[Title/Abstract])) OR (Aerobic Exercises[Title/Abstract])) OR (Exercise Training[Title/Abstract])) OR (Exercise Trainings[Title/Abstract]) OR (Resistance Training[MeSH Terms])) OR (Training, Resistance[Title/Abstract])) OR (Strength Training[Title/Abstract])) OR (Training, Strength[Title/Abstract]))) OR (Weight Lifting Strengthening Program[Title/Abstract])) OR (Exercise Program, Weight Lifting[Title/Abstract])) OR (Weight Lifting Exercise Program[Title/Abstract])) OR (Strengthening Programs, Weight Bearing[Title/Abstract])) OR (Weight Bearing Exercise Program[Title/Abstract])) OR (Endurance Training[MeSH Terms])) OR (Training, Endurance[Title/Abstract])
#3	#1 AND #2

Table S2. Search strategy in Web of Science.

Search	Query
#1	TS=(whey proteins OR whey OR whey protein OR WP OR whey supplementation OR whey protein supplementation OR whey intake OR WPI OR whey protein isolate OR WPC OR whey protein concentrate OR milk proteins OR milk protein OR dairy proteins OR dairy protein)
#2	TS=(Exercise OR Exercises OR Exercise, Physical OR Exercises, Physical OR Physical Activity OR Activities, Physical OR Activity, Physical OR Physical Activities OR Aerobic Exercise OR Aerobic Exercises OR Exercise Training OR Exercise Trainings OR Resistance Training OR Training, Resistance OR Strength Training OR Training, Strength OR Weight-Lifting Strengthening Program OR Exercise Program, Weight-Lifting OR Weight Lifting Exercise Program OR Strengthening Programs, Weight-Bearing OR Weight Bearing Exercise Program OR Endurance Training OR Training, Endurance)
#3	#1 AND #2

Table S3. Search strategy in Embase.

Search	Query
#1	'whey protein'/exp OR 'whey proteins':ab,ti,kw OR whey:ab,ti,kw OR 'whey protein':ab,ti,kw OR wp:ab,ti,kw OR 'whey supplementation':ab,ti,kw OR 'whey protein supplementation':ab,ti,kw OR 'whey intake':ab,ti,kw OR wpi:ab,ti,kw OR 'whey protein isolate':ab,ti,kw OR wpc:ab,ti,kw OR 'whey protein concentrate':ab,ti,kw OR 'milk proteins':ab,ti,kw OR 'milk protein':ab,ti,kw OR 'dairy proteins':ab,ti,kw OR 'dairy protein':ab,ti,kw
#2	'resistance training'/exp OR 'resistance exercise':ab,ti,kw OR 'resistance exercise training':ab,ti,kw OR 'resistance-type exercise':ab,ti,kw OR 'resistance-type training':ab,ti,kw OR 'strength training':ab,ti,kw OR 'strength-type exercise':ab,ti,kw OR 'strength-type training':ab,ti,kw OR 'physical activity':ab,ti,kw OR 'aerobic exercise':ab,ti,kw OR 'weight-lifting strengthening program':ab,ti,kw OR 'exercise program, weight-lifting':ab,ti,kw OR 'weight lifting exercise program':ab,ti,kw OR 'strengthening programs, weight-bearing':ab,ti,kw OR 'weight bearing exercise program':ab,ti,kw OR 'endurance training'/exp OR 'training, endurance':ab,ti,kw
#3	#1 AND #2

Table S4. Search strategy in Scopus.

Search	Query
#1	TITLE-ABS-KEY("whey proteins" OR whey OR "whey protein" OR WP OR "whey supplementation" OR "whey protein supplementation" OR "whey intake" OR WPI OR "whey protein isolate" OR WPC OR "whey protein concentrate" OR "milk proteins" OR "milk protein" OR "dairy proteins" OR "dairy protein")
#2	TITLE-ABS-KEY("exercise" OR "exercises" OR "physical exercise" OR "physical exercises" OR "physical activity" OR "physical activities" OR "aerobic exercise" OR "aerobic exercises" OR "exercise training" OR "exercise trainings" OR "resistance training" OR "training, resistance" OR "strength training" OR "training, strength" OR "weight lifting strengthening program" OR "exercise program, weight lifting" OR "weight lifting exercise program" OR "strengthening programs, weight bearing" OR "weight bearing exercise program" OR "endurance training" OR "training, endurance")
#3	#1 AND #2

Figure S1-S6. Sensitivity analysis

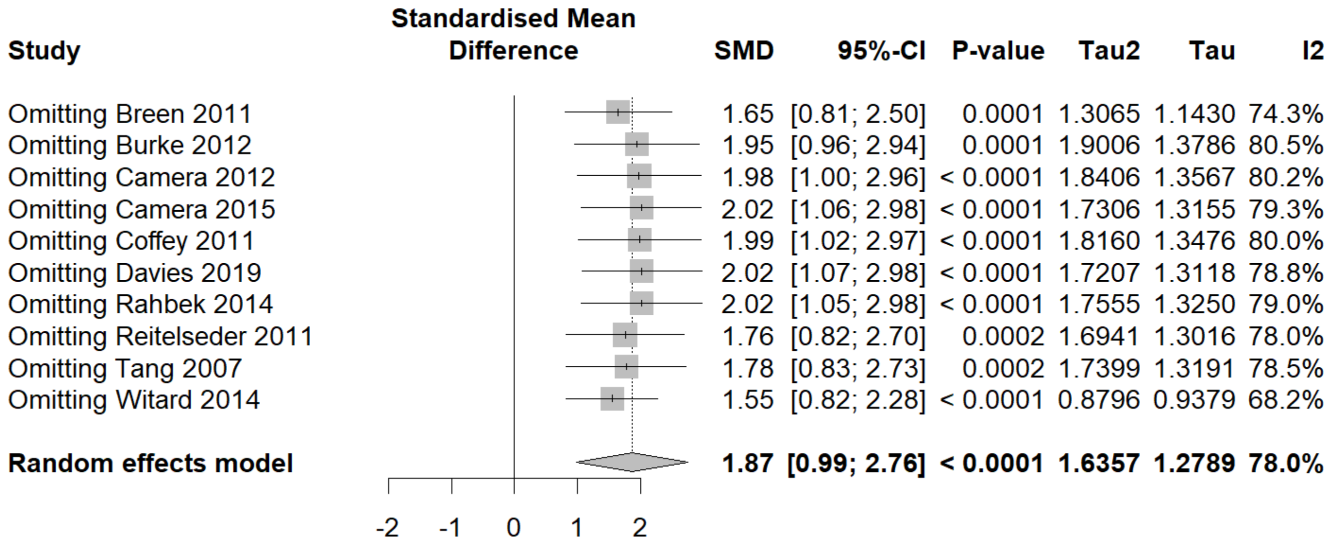

Figure S1. Sensitivity analysis of muscle protein fractional synthetic rates.

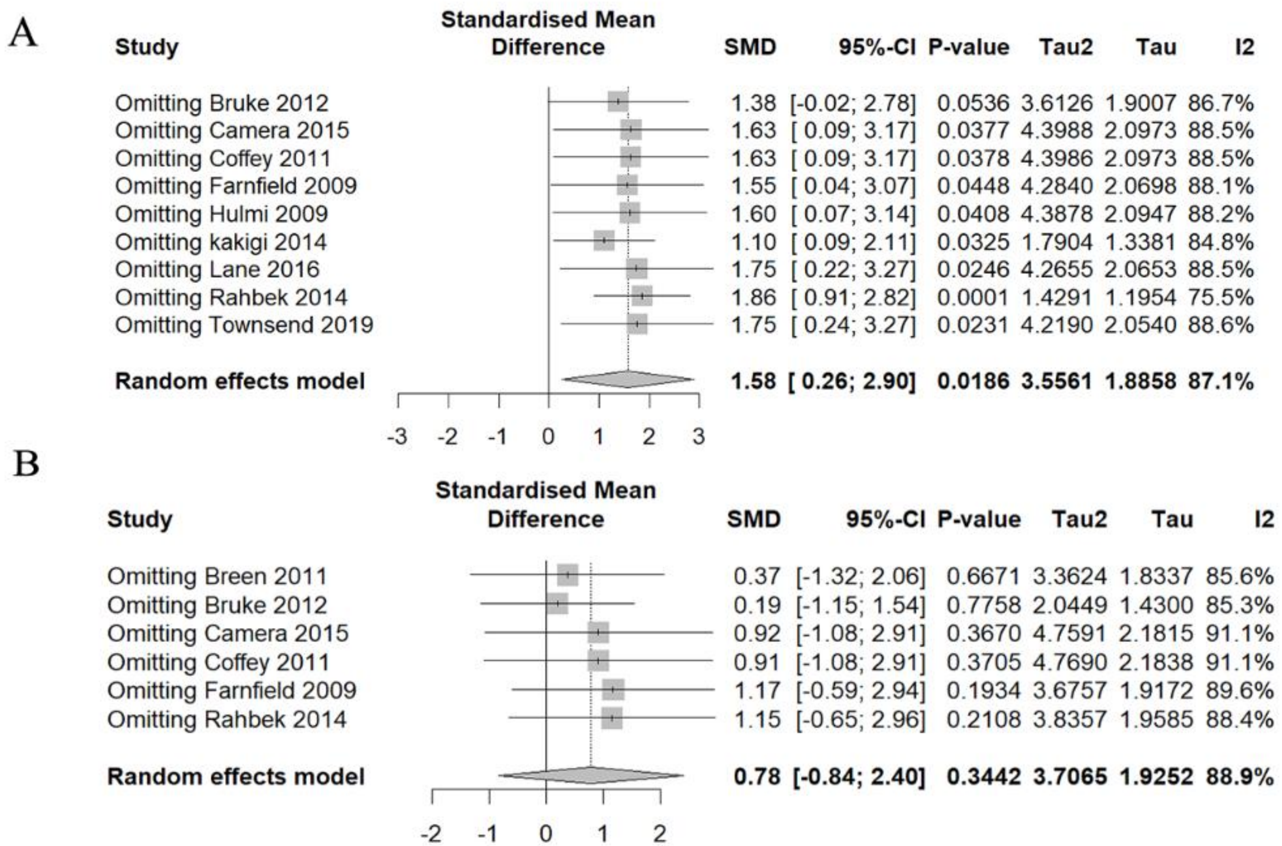

Figure S2. Sensitivity analysis of AKT phosphorylation at 1-2 hours (A), and 4-5 hours (B) post-exercise.

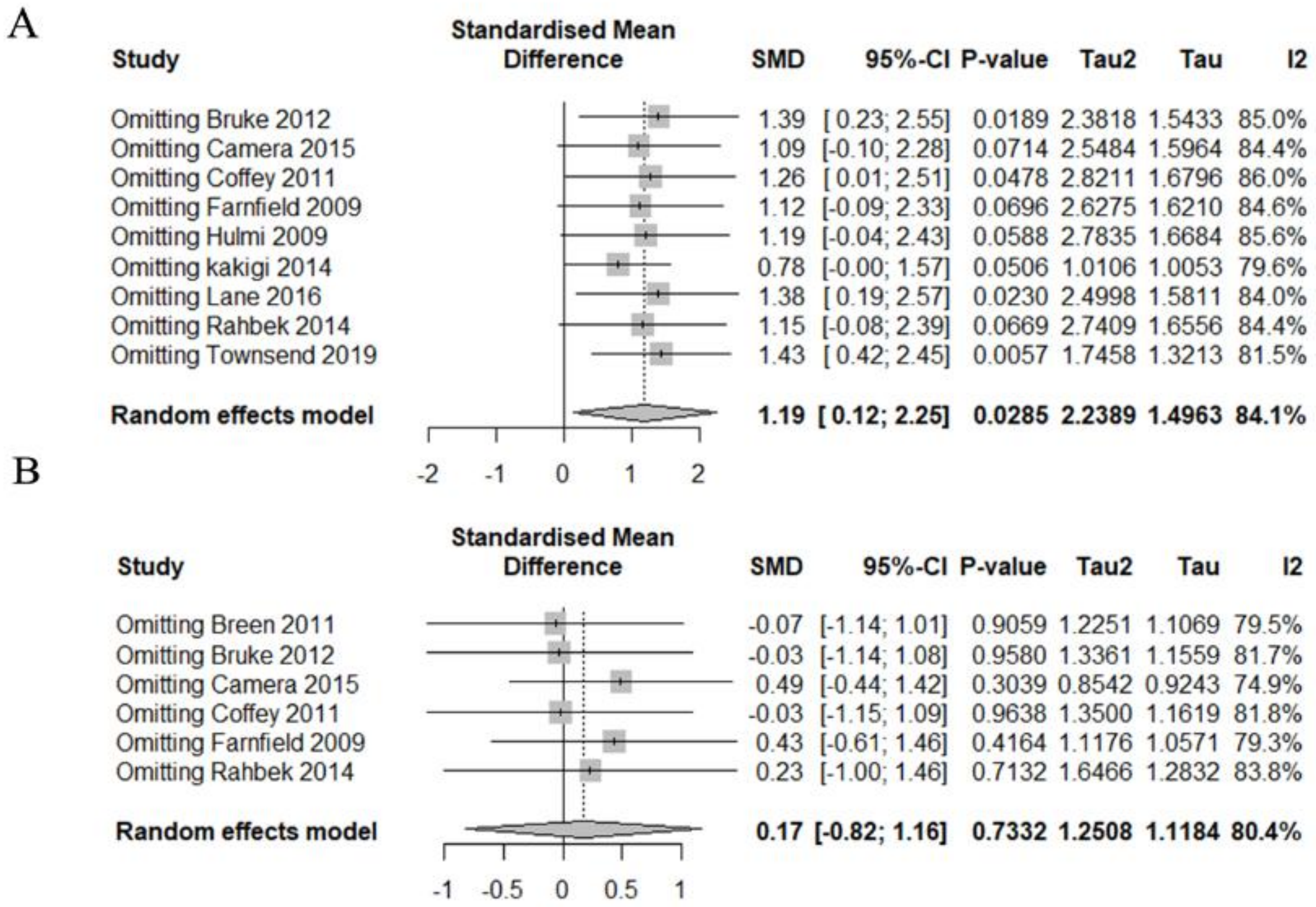

Figure S3. Sensitivity analysis of mTOR phosphorylation at 1-2 hours (A), and 4-5 hours (B) post-exercise.

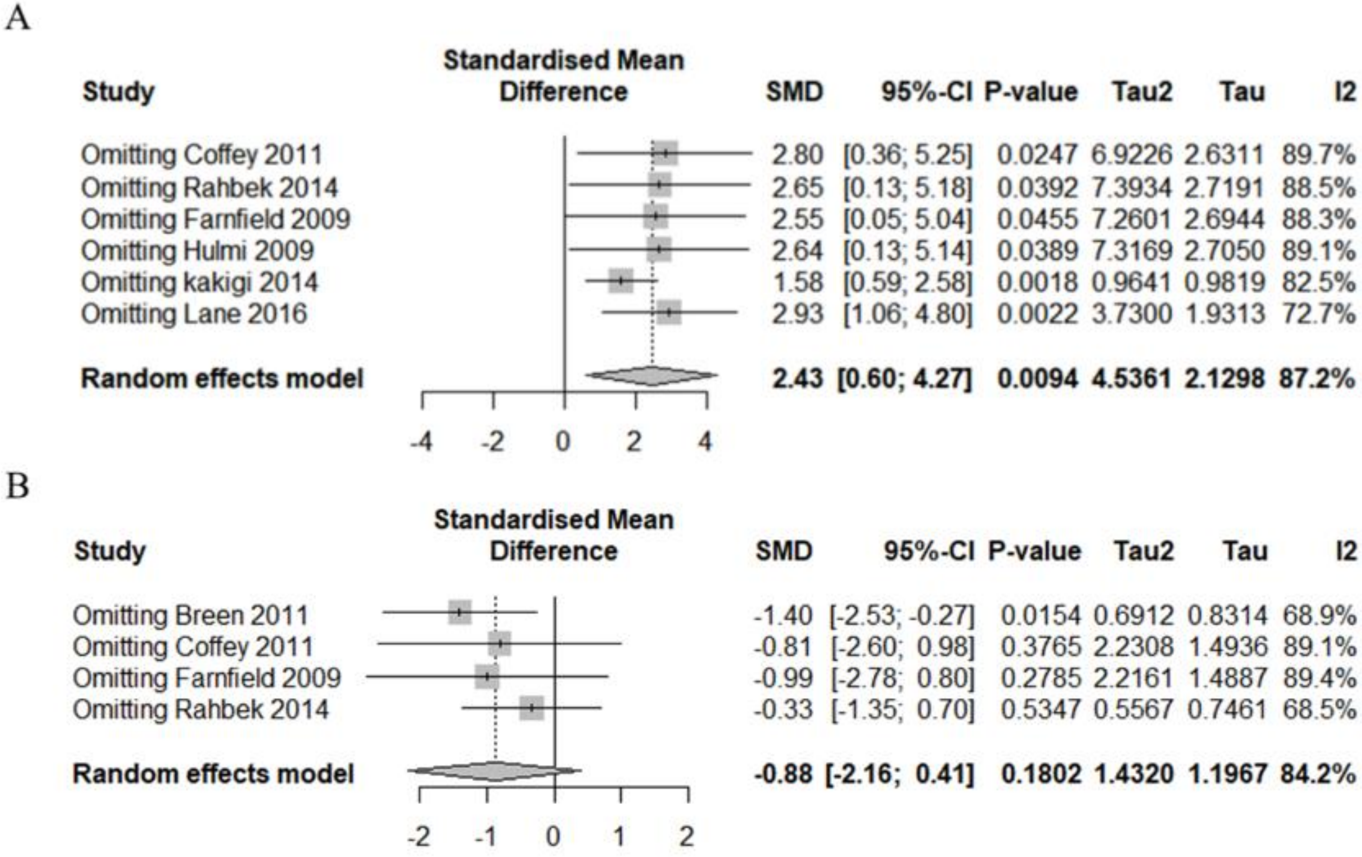

Figure S4. Sensitivity analysis of 4EBP-1 phosphorylation at 1-2 hours (A), and 4-5 hours (B) post-exercise.

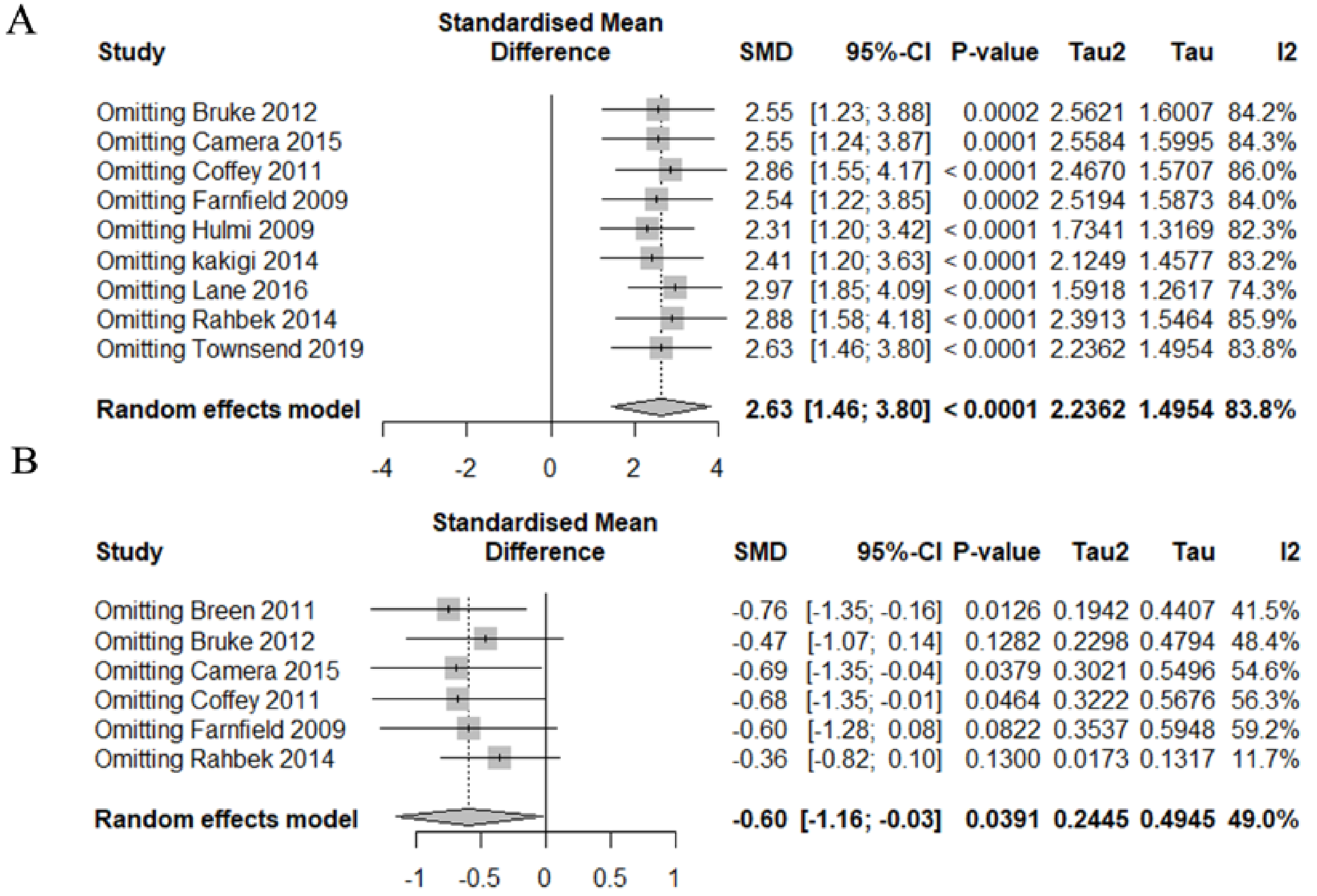

Figure S5. Sensitivity analysis of p70S6K phosphorylation at 1-2 hours (A), and 4-5 hours (B) post-exercise.

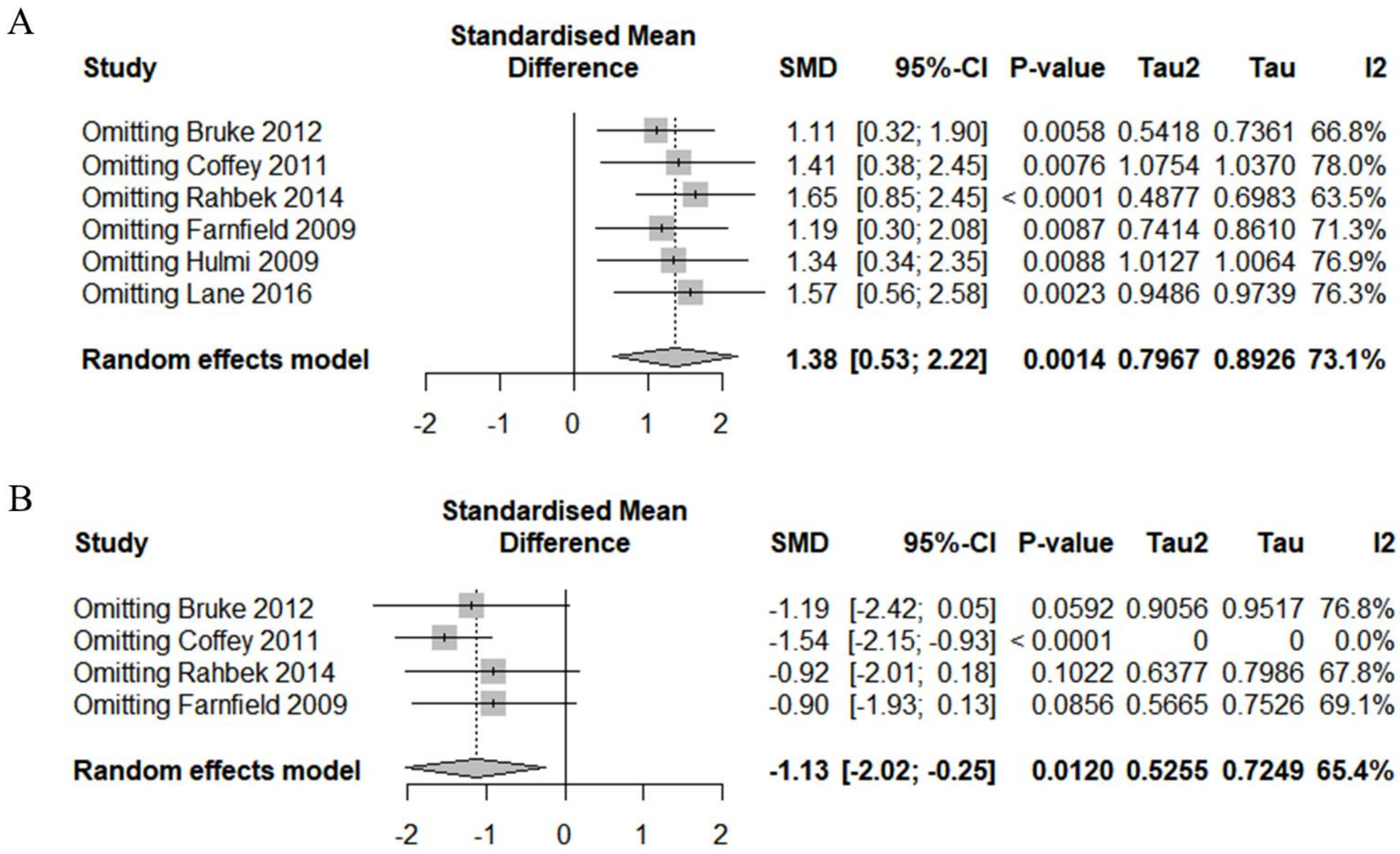

Figure S6. Sensitivity analysis of rpS6 phosphorylation at 1-2 hours (A), and 4-5 hours (B) post-exercise.
